# Brugada phenocopy or congenital Brugada syndrome in a patient with spontaneous pneumopericardium and pericarditis

**DOI:** 10.1002/joa3.12488

**Published:** 2020-12-23

**Authors:** Pichmanil Khmao, Vannak Long, No Ku, Sivutha Lim

**Affiliations:** ^1^ Department of Cardiology and Geriatric Medicine Khmer‐Soviet Friendship Hospital Phnom Penh Cambodia; ^2^ Medical Intensive Care Unit Khmer‐Soviet Friendship Hospital Phnom Penh Cambodia

**Keywords:** Brugada phenocopy, Brugada syndrome, pericarditis, pulmonary tuberculosis, spontaneous pneumopericardium

## Abstract

Brugada syndrome (BrS) is characterized by coved ST segment elevation in the right precordial lead (V1‐V3). Previous reports have described type‐1 or type‐2 Brugada ECG pattern as a Brugada phenocopy (BrP) in various clinical condition and once the etiology is resolved, the BrP ECG pattern normalizes. We describe a case report of type‐1 Brugada ECG pattern in a patient with acquired immunodeficiency syndrome (AIDS) and active pulmonary tuberculosis, which developed to spontaneous pneumopericardium and pericarditis. The coexistence of type‐1 Brugada ECG pattern with spontaneous pneumopericardium and pericarditis is an extremely rare pathological condition that has not been previously described.

## Graphical abstract

Brugada phenocopy or congenital Brugada syndrome in a patient with acquired immunodeficiency syndrome (AIDS) and active pulmonary tuberculosis, which developed to pneumopericardium and pericarditis.

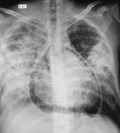

## 1 INTRODUCTION

Brugada phenocopy (BrP) or acquired Brugada syndrome (BrS) is a clinical condition where the ECG pattern mimics to congenital BrS but is secondary to underlying pathological condition. Once the etiology is resolved, the BrP ECG pattern normalizes. Pneumopericardium is defined as an accumulation of air in the pericardial sac. Pneumopericardium usually results from trauma and less commonly in nontraumatic causes including iatrogenic and noniatrogenic. Noniatrogenic etiologies consist of infectious process secondary to gas‐producing bacilli in the pericardial fluid or fistulous communication between pericardium and air‐containing surrounding organs such as bronchus, esophagus, and stomach. A coexistence of type‐1 Brugada ECG pattern in spontaneous pneumopericardium and pericarditis with active pulmonary tuberculosis on a patient with AIDS is an extremely rare clinical scenario. To the best of our knowledge, this is the first case in which these conditions are described.

## 2 CASE REPORT

The patient is a 35‐year‐old male who complained of fever and dyspnea for approximately 6 weeks. He presented a fever of 39.5°C and severe shortness of breath with mild productive cough of purulent sputum for approximately 1 week prior to admission. There was no history of trauma, prior thoracic surgery, or cardiopulmonary disease. He denied recent use of any new medications. There was no history of syncope or family history of sudden cardiac death (SCD).

On clinical examination, he appeared alert but in acute respiratory distress. His blood pressure was 130/85 mm Hg with a pulse rate of 120 beats per minute, respiratory rate of 30 per minute, and temperature of 39.5°C. Chest examination revealed bilateral crackles on pulmonary auscultation. Heart sounds were muffled with no murmurs. There were no peripheral signs of right heart failure. Peripheral pulses were palpable. Abdominal examination was unremarkable.

Laboratory results noted the following abnormalities: leukocyte, 8.59 K/µL with 97% of neutrophils and 2% of lymphocytes; hemoglobin, 8 g/dL; platelets, 505 K/µL; and C‐reactive protein, 1.0 mg/L. Electrolytes (Na^+^: 139 mmol/L, K^+^: 4.2 mmol/L, Magnesium: 1.9 mEq/L), renal, and hepatic function were all within normal ranges. The patient had positive antibodies (Serodia and Combo test) to human immunodeficiency virus (HIV). His sputum was taken for smear test which presented of acid‐fast bacilli (+++) with no pathogen isolated.

**FIGURE 1:**
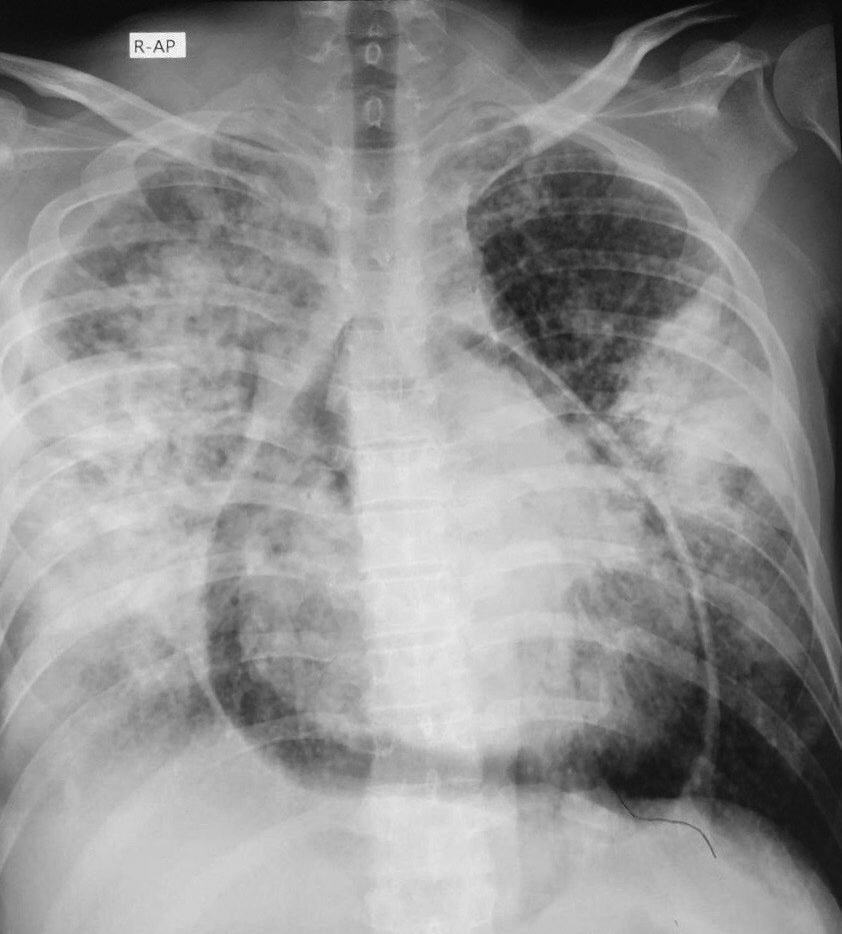
Antero‐posterior chest radiograph shows an air space surrounding the heart consistent with pneumopericardium. In addition, there are bilateral infiltrates in both lung fields

**FIGURE 2:**
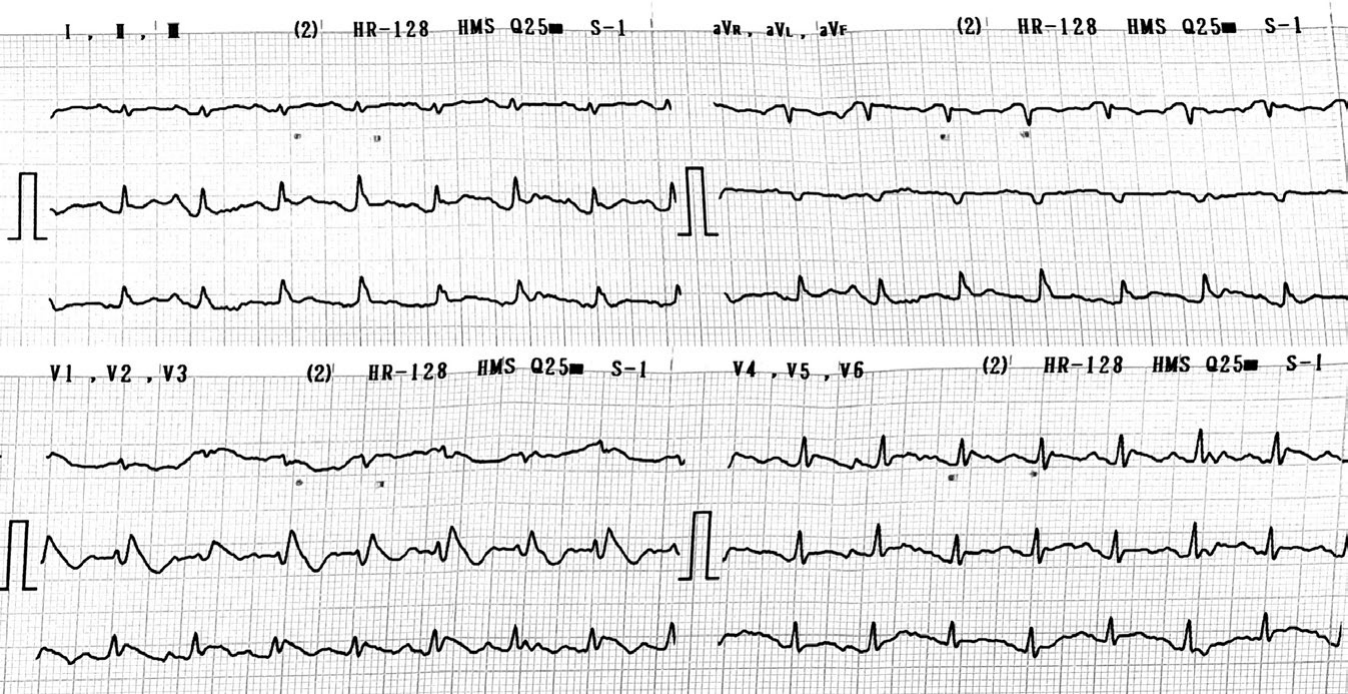
Admission ECG shows coved‐type ST segment elevation in the right precordial leads suggesting type‐1 Brugada ECG pattern. PR segment elevation with ST depression in aVR and widespread PR segment depression in almost all leads consistent with pericarditis

Chest x‐ray revealed a translucent shadow around the cardiac silhouette consistent with pneumopericardium as well as bilateral infiltration and patchy areas of consolidation (Figure 1). ECG showed coved‐type ST segment elevation from V1‐V3 suggesting type‐1 Brugada ECG pattern (Figure 2). In addition, his ECG presented PR segment elevation with ST depression in aVR, subtle concave ST segment elevation and minimal PR segment depression in almost all leads consistent with pericarditis as well as low QRS voltage resulting from pneumopericardium (Figure 2). A bedside transthoracic echocardiography failed to visualize the heart as a consequence of air artifact. Unfortunately, by reason of his financial constraints, a computed tomography (CT) scan of the chest could not be performed. He was treated with antibiotics and antipyretics but left against medical advice and expired within 48 hours because of respiratory distress.

## 3 DISCUSSION

Spontaneous pneumopericardium in a patient with AIDS and active pulmonary tuberculosis is a rare condition and only several cases have been reported. The present case is additionally unusual because type‐1 Brugada ECG pattern and pericarditis ECG changes were also found. In this report, the probable mechanism of pneumopericardium and pericarditis in immunocompromised patient could be a communicating fistula between bronchoalveolar and pericardial cavity as a result of necrotizing pulmonary process by mycobacterium tuberculosis. Manli Yu et al reported a patient with acute pericarditis having type‐1 Brugada ECG pattern which resolved within a few days after treatment of pericarditis.^1^ He described it as a BrP with pericarditis as an underlying cause. Recently, there have been many reports describing the etiologies of BrP such as acute pericarditis^1^, myocardial ischemia,^2^ Takotsubo cardiomyopathy,^3^ and pulmonary embolism.^4^ Contrary to the characteristic of ECG in BrP, the ECG pattern in congenital BrS is often dynamic and sometime may be concealed which could be unmasked by fever, sodium channel blockers, or recording ECG at the higher lead positions.

In our present case, we proposed the following hypotheses as the cause of the type‐1 Brugada ECG pattern: (a) BrP secondary to pericarditis with pneumopericardium or (b) congenital BrS in the setting of pericarditis with pneumopericardium. The presentation of type‐1 Brugada ECG pattern without a history of syncope and family history of SCD along with typical pericarditis changes in the present case could be a “BrP”. Based on the morphologic classification system proposed by Anselm et al,^5^ our patient could be classified as type‐1B BrP. However, we could not obtain follow‐up ECGs or perform a drug challenge because he died shortly after leaving the hospital.

## 4 CONCLUSION

Type‐1 Brugada ECG pattern and spontaneous pneumopericardium with pericarditis have not been described in the past. In the present case, this may be a result from congenital BrS unmasked by fever or may represent a phenocopy secondary to acute pericarditis. Prior clinical symptoms, family history, a positive provocative test, or the finding of underlying provocateurs of the BrP are helpful in making this distinction.

## CONFLICT OF INTEREST

Authors declare no conflict of interests for this article.
